# Prevalence of Locomotive Syndrome in Perioperative Patients With Localized Cancer

**DOI:** 10.7759/cureus.88699

**Published:** 2025-07-24

**Authors:** Yoshimi Katayama, Eiji Nakata, Takuto Itano, Yoshiteru Akezaki, Masanori Hamada, Toshifumi Ozaki

**Affiliations:** 1 Department of Rehabilitation Medicine, Okayama University Hospital, Okayama, JPN; 2 Department of Orthopaedic Surgery, Okayama University Hospital, Okayama, JPN; 3 Division of Physical Therapy, Kochi Professional University of Rehabilitation, Kochi, JPN

**Keywords:** aging, cancer, locomotive syndrome, muscle strength, perioperative system, physical function, risk factors

## Abstract

Introduction

Many patients with cancer experience reduced activities of daily living due to muscle weakness and fatigue caused by underlying symptoms and treatment side effects. However, the incidence of locomotive syndrome, which may reduce mobility due to motor dysfunction in patients with cancer, has not been sufficiently explored. Therefore, we aimed to investigate the incidence of locomotive syndrome and identify its risk factors in perioperative patients with cancer.

Methods

We included 636 perioperative patients with localized cancer who were treated between 2020 and 2023. The severity of locomotive syndrome was classified into stages 1, 2, and 3.

Results

The overall locomotive syndrome rate was 88.1%, with distribution across stages: stage 1 (56.8%), stage 2 (17.5%), and stage 3 (13.8%). Among men, the overall incidence was 86.5%, with stage 1 (60.3%), stage 2 (15.5%), and stage 3 (10.7%). Among women, the overall incidence was 90.6%, with stage 1 (50.6%), stage 2 (20.9%), and stage 3 (19.1%). Half of patients in their 20s and two-thirds in their 30s had locomotive syndrome. The rates were 58.6%, 80.4%, 81.8%, 93.2%, and 97.8% in the 40s, 50s, 60s, 70s, and 80s age groups, respectively. Individuals in their 40s had significantly lower rates than those in older groups. Age, grip strength, and percent vital capacity were identified as risk factors.

Conclusion

A high prevalence of locomotive syndrome was observed among patients with localized cancer. Age, reduced grip strength, and lower respiratory capacity were identified as associated factors. While the findings suggest possible implications for postoperative recovery, further validation through longitudinal studies is required.

## Introduction

As life expectancy for patients with cancer improves, the demand for survivorship services to support ongoing social activities such as work, housework, and hobbies while receiving cancer treatment is increasing [1,2]. Maintaining mobility is crucial for sustaining these activities. However, many patients with cancer experience a decline in activities of daily living due to muscle weakness and fatigue resulting from the underlying disease symptoms and side effects of chemotherapy, radiation therapy, and surgery, compounded by disuse associated with hospitalization [3,4]. These factors can affect the locomotive organs, such as bones, muscles, and nerves, in ways that interfere with movement [5-7]. Locomotive syndrome (LS) manifests in the late middle-aged to older adult population, who are at a high risk of systemic musculoskeletal disability [8,9]. LS results in motor function deterioration and musculoskeletal disorders, which impair health-related quality of life and increase the need for nursing care [10-13]. Furthermore, LS is a risk factor for cardiovascular disease, reduced quality of life, and increased medical costs [14]. Yamada et al. revealed that 59.4% of 8,681 independent community dwellers in Japan had LS [15]. Their study revealed age-dependent deterioration in LS risk test scores for both sexes, with a higher risk associated with a body mass index (BMI) of >25 kg/m^2^ and lack of exercise habits. Yoshimura et al. estimated the prevalence of LS at 69.8% and 25.1% for stages 1 and 2, respectively, in the entire population. They further projected that 45,900,000 and 13,800,000 people aged >40 years could be categorized as having LS stages 1 and 2, respectively [10].

The possibility of LS is higher among older people, as cancer incidence is high in this population. In 2018, the Japanese Orthopedic Association expanded this concept to the field of oncology, identifying “LS in patients with cancer.” The available data are limited, with only two reports on LS, and the reported rates of LS among patients with cancer were 79-96% [5,7]. Sato et al. reported that the proportion of patients with cancer at LS stage 2 was significantly higher than that of those without cancer (51% vs. 13%, p<0.001) [7]. They also reported that patients with cancer had worse scores than those without cancer on functional tests (e.g., stand-up and two-step tests) and the 25-question Geriatric Locomotive Function Scale (GLFS-25), a self-administered questionnaire that assesses motor dysfunction. However, these data have limitations in characterizing outcomes across various stages, and data specifically on perioperative patients with localized cancer are lacking. No study has investigated the incidence rate of preoperative LS in patients with various cancer types. Furthermore, the factors associated with LS have not been sufficiently explored in patients with cancer. Therefore, we aimed to investigate the prevalence of LS and identify associated risk factors in perioperative patients with localized cancer.

## Materials and methods

Study population

We retrospectively evaluated the medical records of patients with several cancer types who underwent surgery at our institution between April 2020 and August 2023. Our target population comprised patients with cancer referred from the perioperative system conducted at the hospital [16]. Therefore, only patients with cancer who underwent invasive surgeries, such as open chest or abdominal surgery, were included. Patients with breast and bladder cancers were excluded. The inclusion criterion was a pathologically proven diagnosis of cancer. Patients with metastases at the first presentation or those treated for metastatic resection were excluded. During this period, 636 patients received treatment for this diagnosis (401 men and 235 women; median age, 72 years [range, 25-94 years]) (Table 1). The primary tumor sites included the lungs (269 patients; median age, 72 years [range, 37-94 years]), esophagus (118 patients; median age, 69 years [range, 42-88 years]), head and neck (81 patients; median age, 70 years [range, 25-89 years]), colorectum (53 patients; median age, 75 years [range, 41-90 years]), pancreas (41 patients; median age, 72 years [range, 42-86 years]), gastric (24 patients; median age, 79.5 years [range, 63-93 years]), and other (50 patients). Patients with gastric cancer were significantly older than those with other cancers, including lung (p<0.01), esophageal (p<0.01), head and neck (p<0.01), colorectal (p<0.05), and pancreatic cancers (p<0.01). Breast and bladder cancer cases were excluded because their surgical procedures are generally less invasive and thus not fully comparable in terms of LS burden.

**Table 1:** Patient characteristics.

Characteristics	Category	Patients, number
Sex	Men	401
	Women	235
Age, year	Median (range)	72 (25–94)
Histology	Lung cancer	269
	Esophageal cancer	118
	Head and neck cancer	81
	Colorectal cancer	53
	Pancreatic cancer	41
	Gastric cancer	24
	Bile duct cancer	19
	Hepatocellular carcinoma	15
	Small bowel cancer	14
	Anal cancer	1
	Gallbladder cancer	1

Measurements of physical function

Grip Strength

Grip strength was measured using a grip strength meter (TKK 5401 Grip-D; Takei, Niigata, Japan). Participants were instructed to grasp the meter with the pointer facing the outward direction. Before measurement, the interphalangeal joints of the fingers were adjusted to nearly right angles. Hand grip strength was measured twice on each side, and the maximum value was recorded. The cutoff values were 28 kg and 18 kg for men and women, respectively [17].

Walking Time

Participants walked along a 10 m path at their usual walking speed. A spare path of 1 m was provided for walking. Measurements of usual walking speed were obtained twice, and the fastest speed was recorded. The cutoff value was 10 s [17].

Assessment of LS Severity

The severity of LS was assessed using the LS risk test, which consists of two functional performance assessments: the stand-up test and the two-step test, as well as the GLFS-25. The validity, reliability, and feasibility of this comprehensive assessment method have been demonstrated in previous studies [8,9].

The stand-up test was used to assess lower limb muscle strength. Participants attempted to stand up from stools of varying heights (40 cm, 30 cm, 20 cm, and 10 cm), initially using both legs. If successful, they then attempted to stand using one leg. Each movement was performed without using momentum, and participants were required to maintain an upright posture for at least 3 s. The lowest seat height from which the participant could successfully stand determined their result. The severity stage was assigned based on this performance, with lower achievable heights and unilateral efforts indicating higher severity.

The two-step test evaluated walking ability by measuring the maximum stride length relative to the participant’s height. Participants were instructed to take two large steps forward starting from a marked line and then bring both feet together. The total distance covered was divided by the participant’s height to calculate the two-step value. The better of two trials was used for scoring. Based on the result, LS severity was classified as stage 0 (≥1.3), stage 1 (1.1-1.29), stage 2 (0.9-1.09), or stage 3 (<0.9).

The GLFS-25 is a self-administered questionnaire designed to evaluate physical function, pain, social interaction, and mental status over the previous month. It consists of 25 items, each rated on a five-point scale from 0 (no impairment) to 4 (severe impairment), with total scores ranging from 0 to 100. Scores were interpreted as follows: <7 indicated stage 0; 7-15, stage 1; 16-23, stage 2; and ≥24, stage 3.

Following completion of all three assessments, each participant was classified into an LS severity stage ranging from stage 0 (no impairment) to stage 3 (most severe). The final stage was determined based on the most severe result among the three tests and was used for all subsequent analyses [5].

All physical function tests, including grip strength, walking speed, and the LS risk tests (stand-up test, two-step test, and GLFS-25), were administered by trained rehabilitation staff following standardized institutional protocols to ensure consistency and reduce inter-rater variability. Pulmonary function tests, including percent vital capacity (%VC) and FEV1, were conducted for all patients in the university hospital’s pulmonary function laboratory under standardized conditions.

Assessment of Study Outcomes

The LS rate was investigated during the initial surgery and was determined in instances where there were ≥20 cases. We assessed the association of the following variables with LS in patients at initial surgery: age, sex, BMI, cancer type, hand grip strength, walking speed, %VC, and percent forced expiratory volume in 1 s (FEV1%).

Statistical analysis

Fisher’s exact test was used for categorical parameters. Univariate analysis was performed using the Fisher’s exact and Mann-Whitney U tests to identify factors associated with LS. Subsequently, logistic regression was used for multivariate analysis. All analyses were performed using IBM SPSS Statistics for Windows, Version 22.0 (Released 2013; IBM Corp., Armonk, New York, USA). All tests were two-sided, and statistical significance was set at p<0.05. Patients with missing data in any of the key assessment variables, including grip strength, walking time, pulmonary function (%VC and FEV1), or components of the LS risk tests (stand-up test, two-step test, GLFS-25), were excluded from the corresponding analyses.

Ethics

The study was conducted in accordance with the Declaration of Helsinki and approved by the relevant ethics committee (approval number: KEN2312-014).

## Results

Two-step test, stand-up test, and the GLFS-25 score in each age category

Table 2 presents the results of the two-step test, stand-up test, and the GLFS-25 score in each age category. The percentage of individuals with the ability to stand up with one leg from a 40-cm-high seat (either leg) (%) and with both legs from a 20-cm-high seat (%) decreased with age. The mean two-step score decreased with age: 1.5, 1.4, 1.4, 1.2, and 1.1 in the 40s, 50s, 60s, 70s, and 80s, respectively. Conversely, the mean GLFS-25 increased with age: 5, 5, 5, 6, and 10 in the 40s, 50s, 60s, 70s, and 80s, respectively. Significant differences were observed by age and sex.

**Table 2:** Prevalence of each stage of the stand-up test by age and median of the two-step test and GLFS-25 by age. GLFS-25: 25-question Geriatric Locomotive Function Scale. ^a^Significantly different (p<0.05) from the values of those aged 60–69. ^b^Significantly different (p<0.05) from the values of those aged 70–79. ^c^Significantly different (p<0.05) from the values of those aged 80–89. ^d^Significantly different (p<0.05) from the values of men.

	40–49 years			50–59 years			60–69 years			70–79 years			80-89 years		
	Men (n=17)	Women (n=12)	Total (n=29)	Men (n=33)	Women (n=23)	Total (n=56)	Men (n=106)	Women (n=53)	Total (n=159)	Men (n=186)	Women (n=107)	Total (n=293)	Men (n=51)	Women (n=38)	Total (n=89)
Ability to stand up with one leg from a 40-cm-high seat (either leg) (%)	58.8^a,b,c^	41.7^b,c^	51.7^a,b,c^	39.4^b,c^	39.1^b,c^	39.3^b,c^	25.5^b,c^	24.5^b,c^	25.2^b,c^	14.5	8.4	12.3	9.8	0	5.6
Ability to stand up with both legs from a 20-cm-high seat (%)	94.1	100^c^	96.6^c^	93.9	91.3^a,c^	92.9^c^	91.5	90.6^c^	91.2^c^	90.9	83.2^c^	88.1^c^	86.3	55.3^d^	73
Ability to stand up with both legs from a 30-cm-high seat (%)	94.1	100	96.6	100	91.3	96.4	97.2	100^ c^	98.1^c^	97.3	7.5^c^	95.6^c^	98	76.3^d^	88.8
Two-step test	1.5^a,b,c^	1.4^b,c^	1.5^a,b,c^	1.5^a,b,c^	1.4^a,b,c^	1.4^a,b,c^	1.4^b,c^	1.3^b,c,d^	1.4^b,c^	1.3^c^	1.2^c,d^	1.2^c^	1.1	1.1^d^	1.1
	(1.0–1.7)	(1.1–1.5)	(1.0–1.7)	(1–1.7)	(0–1.7)	(0–1.7)	(0.9–1.7)	(0.8–1.6)	(0.8–1.7)	(0–1.7)	(0–1.5)	(0–1.7)	(0­–1.5)	(0–1.3)	(0–1.5)
25-Question GLFS	5 (0–39)	4.5 (0–16)^c^	5 (0–39)^c^	5 (0–26)^c^	5 (0–21)^b,c^	5 (0–26)^c^	4 (0–69)^c^	6 (0–32)^c^	5 (0–69)^c^	5 (0–54)^c^	8 (0–54)^d^	6 (0–54)^c^	10 (0–57)	11 (0–56)	10 (0–57)

LS in preoperative patients with localized cancer in each LS risk test

Table 3 shows the age and sex distributions of each LS risk test. The rate of LS determined using the stand-up test was 80.8%, with stages 1 (68.1%), 2 (8.0%), and 3 (4.7%). Significant differences were observed by age and sex. The rate of LS determined using the two-step test was 53.8%, with stages 1 (36.3%), 2 (11.5%), and 3 (6.0%). Significant differences were observed by age and sex. The rate of LS determined using the GLFS-25 was 47.5%, with stages 1 (29.1%), 2 (9.4%), and 3 (9.0%). Significant differences were observed by age and sex.

**Table 3:** Number of people with LS for each of the three tests by age group. GLFS-25: 25-question Geriatric Locomotive Function Scale; LS: locomotive syndrome. Values are presented as number (%). *Within 20 cases, % not extracted. ^a^Significantly different (p<0.05) from the values of those aged 60–69. ^b^Significantly different (p<0.05) from the values of those aged 70–79. ^c^Significantly different (p<0.05) from the values of those aged 80–89. ^d^Significantly different (p<0.05) from the values of men.

		20–29 years			30–39 years			40–49 years			50–59 years			60–69 years			70–79 years			80–89 years			90>= years			Total		
Items	Stage	Men	Women	Total	Men	Women	Total	Men	Women	Total	Men	Women	Total	Men	Women	Total	Men	Women	Total	Men	Women	Total	Men	Women	Total	Men	Women	Total
Stand-up test	Normal	2 (*)	0	2 (*)	2 (*)	0	2 (*)	10 (58.8)	5 (41.7)	15 (51.7)	13 (39.4)	9 (39.1)	22 (39.3)	27 (25.5)	13 (24.5)	40 (25.2)	27 (14.5)	9 (8.4)	36 (12.3)	5 (9.8)	0	5 (5.6)	0	0	0	86 (21.5)	36 (15.3)	122 (19.2)
	Total	0	0	0	1 (*)	0	1 (*)	7 (41.2)^a,b,c^	7 (58.3)^b,c^	14 (48.3)^a,b,c^	20 (60.6)^b,c^	14 (60.9)^b,c^	34 (60.7)^b,c^	79 (74.5)^b,c^	40 (75.5)^b,c^	119 (74.8)^b,c^	159 (85.5)	98 (91.6)	257 (87.7)	46 (90.2)	38 (100)	84 (94.4)	3 (*)	2 (*)	5 (*)	315 (78.5)	199 (84.7)	514 (80.8)
	Stage 1	0	0	0	1 (*)	0	1 (*)	6 (35.3)	7 (58.3)	13 (44.8)	18 (54.5)	12 (52.2)	30 (53.5)	70 (66)	35 (66)	105 (66.0)	142 (76.3)	80 (74.8)	222 (75.8)	39 (76.5)	21 (55.3)	60 (67.4)	2 (*)	0	2 (*)	278 (69.3)	155 (66.0)	433 (68.1)
	Stage 2	0	0	0	0	0	0	0	0	0	2 (6.1)	0	2 (3.6)	6 (5.7)	5 (9.5)	11 (6.9)	12 (6.5)	10 (9.3)	22 (7.5)	6 (11.8)	8 (21.0)	14 (15.8)	1 (*)	1 (*)	2 (*)	27 (6.7)	24 (10.2)	51 (8.0)
	Stage 3	0	0	0	0	0	0	1 (5.9)	0	1 (3.5)	0	2 (8.7)	2 (3.6)	3 (2.8)	0	3 (1.9)	5 (2.7)	8 (7.5)	13 (4.4)	1 (1.9)	9 (23.7)	10 (11.2)	0	1 (*)	1 (*)	10 (2.5)	20 (8.5)	30 (4.7)
Two-step test	Normal	1 (*)	0	1 (*)	2 (*)	0	2(*)	14 (82.3)	10 (83.4)	24 (82.8)	28 (84.9)	16 (69.6)	44 (78.6)	70 (66)	29 (54.7)	99 (62.3)	83 (44.6)	31 (29.0)	114 (38.9)	9 (17.6)	1 (2.6)	10 (11.2)	0	0	0	207 (51.6)	87 (37.1)	294 (46.2)
	Total	1 (*)	0	1 (*)	1 (*)	0	1(*)	3 (17.7)^b,c^	2 (16.6)^b,c^	5 (17.2)^a,b,c^	5 (15.1)^a,b,c^	7 (30.4)^b,c^	12 (21.4)^a,b,c^	36 (34.0)^b,c^	24 (45.3)^b,c^	60 (37.7)^b,c^	103 (55.4)^c^	76 (71.0)^c,d^	179 (61.1)^c^	42 (82.4)	37 (97.4)	79 (88.8)	3 (*)	2 (*)	5 (*)	194 (48.4)	148 (62.9)	342 (53.8)
	Stage 1	1 (*)	0	1 (*)	1 (*)	0	1(*)	1 (5.9)	1 (8.3)	2 (6.9)	4 (12.1)	6 (26.1)	10 (17.8)	28 (26.4)	20 (37.7)	48 (30.2)	78 (41.9)	53 (49.5)	131 (44.7)	19 (37.3)	18 (47.4)	37 (41.6)	0	1 (*)	1 (*)	132 (32.9)	99 (42.1)	231 (36.3)
	Stage 2	0	0	0	0	0	0	2 (11.8)	1 (8.3)	3 (10.3)	1 (3)	0	1 (1.8)	8 (7.6)	3 (5.7)	11 (6.9)	16 (8.6)	16 (15.0)	32 (10.9)	17 (33.3)	8 (21.1)	25 (28.1)	1 (*)	0	1 (*)	45 (11.2)	28 (11.9)	73 (11.5)
	Stage 3	0	0	0	0	0	0	0	0	0	0	1 (4.3)	1 (1.8)	0	1 (1.9)	1 (0.6)	9 (4.9)	7 (6.5)	16 (5.5)	6 (11.8)	11 (28.9)	17 (19.1)	2 (*)	1 (*)	3 (*)	17 (4.3)	21 (8.9)	38 (6.0)
GLFS-25	Normal	2 (*)	0	2 (*)	2 (*)	0	2(*)	11 (64.7)	7 (58.3)	18 (62.1)	19 (57.6)	13 (56.5)	32 (57.1)	68 (64.2)	28 (52.8)	96 (60.4)	107 (57.5)	47 (43.9)	154 (52.6)	19 (37.3)	11 (28.9)	30 (33.7)	0	0	0	228 (56.9)	106 (45.1)	334 (52.5)
	Total	0	0	0	1 (*)	0	1(*)	6 (35.3)	5 (41.7)	11 (37.9)^c^	14 (42.4)	10 (43.5)	24 (42.9)^c^	38 (35.8)^c^	25 (47.2)^c^	63 (39.6)^c^	79 (42.5)^c^	60 (56.1)^d^	139 (47.4)^c^	32 (62.7)	27 (71.1)	59 (66.3)	3 (*)	2 (*)	5 (*)	173 (43.1)	129 (54.9)	302 (47.5)
	Stage 1	0	0	0	1 (*)	0	1(*)	5 (29.4)	3 (25)	8 (27.6)	9 (27.3)	7 (30.4)	16 (28.6)	26 (24.5)	13 (24.5)	39 (24.5)	51 (27.4)	35 (32.7)	86 (29.4)	17 (33.3)	15 (39.5)	32 (36.0)	2 (*)	1 (*)	3 (*)	111 (27.7)	74 (31.5)	185 (29.1)
	Stage 2	0	0	0	0	0	0	0	2 (16.7)	2 (6.9)	4 (12.1)	3 (13.1)	7 (12.5)	7 (6.6)	7 (13.2)	14 (8.8)	13 (7.0)	10 (9.4)	23 (7.8)	7 (13.7)	7 (18.4)	14 (15.7)	0	0	0	31 (7.7)	29 (12.3)	60 (9.4)
	Stage 3	0	0	0	0	0	0	1 (5.9)	0	1 (3.4)	1 (3)	0	1 (1.8)	5 (4.7)	5 (9.5)	10 (6.3)	15 (8.1)	15 (14.0)	30 (10.2)	8 (15.7)	5 (13.2)	13 (14.6)	1 (*)	1 (*)	2 (*)	31 (7.7)	26 (11.1)	57 (9.0)

LS in preoperative patients with localized cancer

**Figure 1:**
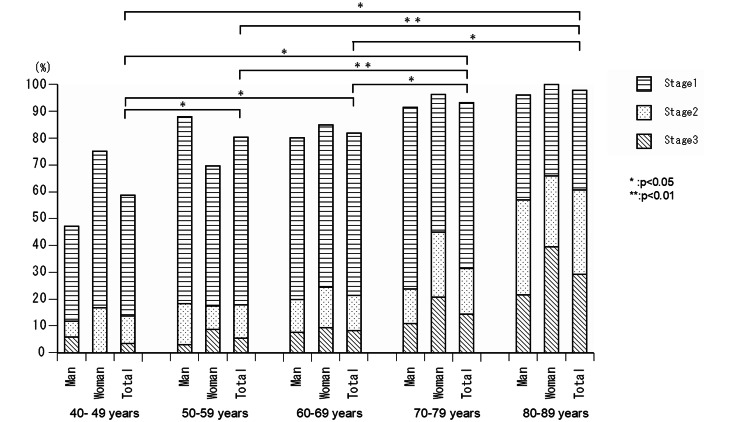
LS in preoperative patients by age. The rates of LS were 58.6%, 80.4%, 81.8%, 93.2%, and 97.8% in the 40s, 50s, 60s, 70s, and 80s, respectively. The rates of LS among men and overall population in their 40s were significantly lower than in those in their 50s, 60s, 70s, and 80s, but only higher than in women in their 70s and 80s (p<0.05). The rates of LS among women and both sexes in their 50s were significantly lower than in those in their 70s and 80s (p < 0.01). The rates of LS among men, women, and both sexes in their 60s were significantly lower than in those in their 70s and 80s (p<0.05). LS: locomotive syndrome.

Table 4 shows the age and sex distributions of overall LS (Figure 1). The overall rate of LS was 88.1%, with stages 1 (56.8%), 2 (17.5%), and 3 (13.8%) among all patients. Among men, the rate of LS was 86.5%, with stages 1 (60.3%), 2 (15.5%), and 3 (10.7%). The rate of LS was 90.6%, with stages 1 (50.6%), 2 (20.9%), and 3 (19.1%) among women. Half of patients in their 20s and two-thirds in their 30s had LS. The rates of LS were 58.6%, 80.4%, 81.8%, 93.2%, and 97.8% in the 40s, 50s, 60s, 70s, and 80s age groups, respectively. The rates of LS among men and the overall population in their 40s were significantly lower than those in their 50s, 60s, 70s, and 80s but only higher than those of women in their 70s and 80s (p<0.05). The rates of LS among women and both sexes in their 50s were significantly lower than those in their 70s and 80s (p<0.01). The rates of LS among men, women, and both sexes combined in their 60s were significantly lower than those in their 70s and 80s (p<0.05).

**Table 4:** Number of people with LS for total assessment by age group. LS: locomotive syndrome. ^*^Within 20 cases, % not extracted. ^a^Significantly different (p<0.05) from the values of those aged 50–59. ^b^Significantly different (p<0.05) from the values of those aged 60–69. ^c^Significantly different (p<0.05) from the values of those aged 70–79. ^d^Significantly different (p<0.05) from the values of those aged 80–89.

	20–29 years			30–39 years			40–49 years			50–59 years			60–69 years			70–69 years			80–89 years			≥90			Total		
Stage	Men	Women	Total	Men	Women	Total	Men	Women	Total	Men	Women	Total	Men	Women	Total	Men	Women	Total	Men	Women	Total	Men	Women	Total	Men	Women	Total
Normal	1 (*)	0	1 (*)	1 (*)	0	1 (*)	9 (52.9)	3 (25.0)	12 (41.4)	4 (12.1)	7 (30.4)	11 (19.6)	21 (19.8)	8 (15.1)	29 (18.2)	16 (8.6)	4 (3.7)	20 (6.8)	2 (3.9)	0	2 (2.2)	0	0	0	54 (13.5)	22 (9.4)	76 (11.9)
Total	1 (*)	0	1 (*)	2 (*)	0	2 (*)	8 (47.1)^a,b,c,d^	9 (75.0)^c,d^	17 (58.6)^a,b,c,d^	29 (87.9)	160 (69.6)^c,d^	45 (80.4)^c,d^	85 (80.2)^c,d^	45 (84.9)^c,d^	130 (81.8)^c,d^	170 (91.4)	103 (96.3)	273 (93.2)	49 (96.1)	38 (100)	87 (97.8)	3 (*)	2 (*)	5 (*)	347 (86.5)	213 (90.6)	560 (88.1)
Stage 1	1 (*)	0 (*)	1 (*)	2 (*)	0	2 (*)	6 (35.3)	7 (58.3)	13 (44.8)	23 (69.7)	12 (52.2)	35 (62.5)	64 (60.4)	32 (60.4)	96 (60.4)	126 (67.7)	55 (51.4)	181 (61.8)	20 (39.2)	13 (34.2)	33 (37.1)	3 (*)	0	3 (*)	242 (60.3)	119 (50.6)	361 (56.8)
Stage 2	0	0	0	0	0	0	1 (5.9)	2 (16.7)	3 (10.3)	5 (15.2)	2 (8.7)	7 (12.5)	13 (12.3)	8 (15.1)	21 (13.2)	24 (12.9)	26 (24.3)	50 (17.1)	18 (35.3)	10 (26.3)	28 (31.5)	0	1 (*)	1 (*)	62 (15.5)	49 (20.9)	111 (17.5)
Stage 3	0	0	0	0	0	0	1 (5.9)	0	1 (3.5)	1 (3.0)	2 (8.7)	3 (5.4)	8 (7.5)	5 (9.4)	13 (8.2)	20 (10.8)	22 (20.6)	42 (14.3)	11 (21.6)	15 (39.5)	26 (29.2)	0	1 (*)	1 (*)	43 (10.7)	45 (19.1)	88 (13.8)

LS in preoperative patients in each cancer

Table 5 shows the overall rate of LS. When ≥20 cases were examined, the overall rates of LS for each cancer were 88.8%, 88.1%, 90.1%, 90.6%, 82.9%, and 95.8% for lung, esophageal, head and neck, colorectal, pancreatic, and gastric cancers, respectively. Gastric cancer had the highest rate of LS (95.8%), while pancreatic cancer exhibited the lowest rate (82.9%), with no significant differences observed. The overall combined proportion of stages 2 and 3 cancers was 58.3% for gastric cancer, which was significantly higher than that of other cancers: lung (32.7%, p=0.01), esophageal (22.9%, p=0.001), head and neck (24.7%, p=0.003), and pancreatic (31.7%, p=0.04) cancer. Among women, the incidence of gastric cancer was 85.7%, which was significantly higher than that of lung (41.6%, p=0.04), esophageal (32.2%, p=0.03), head and neck (33.3%, p=0.03), colorectal (36.0%, p=0.04), and pancreatic (36.4%, p=0.03) cancers. In men, the incidence of gastric cancer was 57.0%, with only esophageal cancer (20.0%, p=0.03) being significantly different. Patients with gastric cancer are older than those with other cancer types.

**Table 5:** Number of people with LS for total assessment LS in each cancer type. LS: locomotive syndrome. Values are presented as number (%). ^a^Patients significantly younger than those with gastric cancer.

Stage	Lung cancer			Esophageal cancer			Head and Neck cancer			Colorectal cancer			Pancreatic cancer			Gastric cancer			Bile duct cancer			Hepatocellular carcinoma			Small bowel cancer			Anal cancer		Gallbladder cancer	
Age, median (range)	72 (37–94)^a^			69 (42–88)^a^			70 (25–89)^a^			75 (41–90)^a^			72 (42–86)^a^			79.5 (63–93)			73 (52–82)			72 (51–90)			72.5 (42–84)			68		70	
	Men	Women	Total	Men	Women	Total	Men	Women	Total	Men	Women	Total	Men	Women	Total	Male	Women	Total	Men	Women	Total	Men	Women	Total	Men	Women	Total	Men	Total	Women	Total
Normal	18 (11.5)	12 (10.6)	30 (11.2)	12 (13.3)	2 (7.1)	14 (11.9)	8 (13.3)	0 (0)	8 (9.9)	5 (16.1)	0 (0)	5 (9.4)	3 (18.8)	4 (16.0)	7 (17.1)	1 (5.9)	0 (0)	1 (4.2)	3 (30.0)	3 (33.3)	6 (31.6)	0 (0)	1 (20.0)	1 (6.7)	3 (30.0)	0 (0)	3 (21.4)	1 (100)	1 (100)	0 (0)	0 (0)
Total	138 (88.5)	101 (89.4)	239 (88.8)	78 (86.7)	26 (92.9)	104 (88.1)	52 (86.7)	21 (100)	73 (90.1)	26 (83.9)	22 (100)	48 (90.6)	13 (81.2)	21 (84.0)	34 (82.9)	16 (94.1)	7 (100)	23 (95.8)	7 (70.0)	6 (66.7)	13 (68.4)	10 (100)	4 (80.0)	14 (93.3)	7 (70.0)	4 (100)	11 (78.6)	0 (0)	0 (0)	1 (100)	1 (100)
Stage 1	97 (62.2)	54 (47.8)	151 (56.1)	60 (66.7)	17 (60.7)	77 (65.2)	39 (65.0)	14 (66.7)	53 (65.4)	15 (48.4)	14 (63.6)	29 (54.7)	9 (56.2)	12 (48.0)	21 (51.2)	8 (47.1)	1 (14.3)	9 (37.5)	5 (50.0)	1 (11.1)	6 (31.6)	5 (50.0)	2 (40.0)	7 (46.7)	4 (40.0)	3 (75.0)	7 (50.0)	0 (0)	0 (0)	1 (100)	1 (100)
Stage 2	25 (16)	26 (23)	51 (19.0)	12 (13.3)	1 (3.6)	13 (11.0)	7 (11.7)	4 (19.0)	11 (13.6)	7 (22.6)	4 (18.2)	11 (20.8)	2 (12.5)	5 (20.0)	7 (17.1)	4 (23.5)	4 (57.1)	8 (33.3)	2 (20.0)	3 (33.3)	5 (26.3)	1 (10.0)	1 (20.0)	2 (13.3)	2 (20.0)	1 (25.0)	3 (21.4)	0 (0)	0 (0)	0 (0)	0 (0)
Stage 3	16 (10.3)	21 (18.6)	37 (13.7)	6 (6.7)	8 (28.6)	14 (11.9)	6 (10.0)	3 (14.3)	9 (11.1)	4 (12.9)	4 (18.2)	8 (15.1)	2 (12.5)	4 (16.0)	6 (14.6)	4 (23.5)	2 (28.6)	6 (25.0)	0 (0)	2 (22.3)	2 (10.5)	4 (40.0)	1 (20.0)	5 (33.3)	1 (10.0)	0 (0)	1 (7.2)	0 (0)	0 (0)	0 (0)	0 (0)

Associated factors of preoperative LS

Univariate analysis identified age (≥60), 10-m walking time, grip strength, and %VC as factors associated with preoperative LS (Table 6). Multivariate analysis revealed that age (≥ 60), grip strength, and %VC (<80％) were the factors associated with preoperative LS (Table 7). The rates of LS were 72% and 91% in patients aged <60 and ≥60, respectively. The rates of LS were 99% and 86% in patients below and within the grip strength criteria, respectively. The rates of LS were 99% and 87% in patients with <80% and ≥80% of %VC, respectively.

**Table 6:** Univariate analysis. BMI: body mass index; %VC: percent vital capacity; FEV1%: percent forced expiratory volume in 1 s.

Variables		Patients, number		p-value
		LS group, n (%)	Non-LS group, n (%)	
Age	<60 years	65 (72.2)	25 (27.8)	<0.0001
	≥60 years	495 (90.7)	51 (9.3)	
Sex	Men	347 (86.5)	54 (13.5)	0.13
	Women	213 (90.6)	22 (9.4)	
BMI	<18 kg/m^2^	73 (93.6)	5 (6.4)	0.135
	≥18 kg/m^2^	487 (87.3)	71 (12.7)	
10 m walking time	>10 s	83 (98.8)	1 (1.2)	<0.0001
	≤10 s	477 (86.4)	75 (13.6)	
Hand grip	Men: <28 kg	116 (99.1)	1 (0.9)	<0.0001
	Women: <18 kg			
	Men: ≥28 kg	444 (85.5)	75 (14.5)	
	Women: ≥18 kg			
%VC	<80%	75 (98.7)	1 (1.3)	0.001
	≥80%	485 (86.6)	75 (13.4)	
FEV1%	<70%	116 (88.5)	15 (11.5)	1
	≥70%	444 (87.9)	61 (12.1)	
Medical history of cancer	Yes	158 (89.8)	18 (10.2)	0.495
	No	402 (87.4)	58 (12.6)	
Cancer recurrence	Yes	61 (87.1)	9 (12.9)	0.845
	No	499 (88.2)	67 (11.8)	
Non-smoking period	>10 years	154 (85.1)	27 (14.9)	0.456
	≤10 years	184 (88.0)	25 (12.0)	
Brinkman index	≥600	214 (89.9)	24 (10.1)	0.312
	<600	346 (86.9)	52 (13.1)	

**Table 7:** Logistic regression analyses. BMI: body mass index; CI, confidence interval; %VC, percent vital capacity. Bold indicates significant difference (p<0.05).

Variables	Odds ratio (95% CI)	p-value
Age (years)	3.095 (1.752–5.469)	<0.0001
Sex	0.640 (0.369–1.112)	0.114
BMI (kg/m²)	2.003 (0.745–5.383)	0.168
10 m walking time (s)	6.679 (0.897–49.726)	0.064
Hand grip (kg)	12.947 (1.760–95.233)	0.012
%VC (%)	10.844 (1.454–80.855)	0.02

## Discussion

We discovered that the overall rate of LS was 88.1%, with 56.8%, 17.5%, and 13.8% for stages 1, 2, and 3, respectively, among patients with localized cancer. Risk factors associated with LS included age (≥60), grip strength, and %VC. When analyzing LS stages 2 and 3, the overall rate of LS was significantly higher for gastric cancer (58.3%) than for other cancers, including lung, esophageal, head and neck, and pancreatic cancer.

Large nationwide studies in Japan have investigated the rate of LS in community dwellers [15,18,19]. Yoshimura et al. investigated 2,177 independent community dwellers with an average age of 42.2 years from seven administrative areas of Japan [15]. They utilized the Locomonitor application, a newly developed remote platform, and revealed that 59.4% of patients had LS. Moreover, LS risk test scores showed age-dependent deterioration in both sexes.

Kobayashi et al. investigated the rate of LS in a community-dwelling cohort [18]. They reported that 35% of older adults aged >40 years who attended public health checkups as part of the Yakumo Study exhibited LS. Yoshimura et al. conducted a prospective, longitudinal study called Research on Osteoarthritis/Osteoporosis Against Disability study [10]. They demonstrated that LS prevalence was estimated at 69.8% and 25.1% for stages 1 and 2, respectively, in the entire population. The prevalence tended to be significantly higher with age, but no significant difference was observed between the sexes.

The rate of LS in patients with cancer at various stages was reported as 79-96%, which is higher than that of community dwellers [5,7]. The reference values for the LS risk test determined in this study were comparable with those reported among independent community dwellers [15] and better than those observed in a cohort study [19]. The discrepancy could be attributed to differences in the study population between patients with cancer and community dwellers and cohorts.

Hirahata et al. reported an overall LS rate of 96.0%, with 33.5%, 21.6%, and 40.9% for stages 1, 2, and 3, respectively, in patients with cancers of various origins and stages [5]. Based on the test type, the rates of LS were 64.8% for the stand-up test (40.9%, 8.0%, and 15.9% for stages 1, 2, and 3, respectively), 77.3% for the two-step test (39.8%, 16.5%, and 21.0% for stages 1, 2, and 3, respectively), and 75.0% for the GLFS-25 (30.1%, 16.5%, and 28.4% for stages 1, 2, and 3, respectively). In line with a previous study, a high overall rate of LS was detected in this study, with an overall LS rate of 88.1% (80.8% for the stand-up test, 53.8% for the two-step test, and 47.5% for GLFS-25). However, relatively mild LS was reported in this study, with stages 1 (56.8%), 2 (17.5%), and 3 (13.8%) among all patients. This finding can be explained by the fact that our cases were restricted to localized cancers. Furthermore, we discovered that LS could start as early as the 20s to 40s in patients with cancer. Therefore, physicians should be careful about the occurrence of LS, even in relatively young patients with malignancies.

The prevalence of LS was reported as 4.6-8.4% in the 40s, 7.8-9.2% in the 50s, 8.3-12.0% in the 60s, and 16.3-24.5% in the 70s in a nationwide cross-sectional questionnaire survey with 9,028 and 303,408 individuals utilizing GLFS-25 [20,21]. In this study, the LS rate was 58.6% in the 40s, 69.6% in the 50s, 84.9% in the 60s, and 93.2% in the 70s, revealing that patients with cancer have a higher rate of LS than that in the dwelling cohort.

The rate of LS among men and both sexes in their 40s was significantly lower than that for those in their 50s, 60s, 70s, and 80s, but higher among women in their 50s and 60s. The rates of LS among women and both sexes combined in their 50s were significantly lower than those in their 70s and 80s. The rates of LS among men, women, and both sexes in their 60s were significantly lower than those in their 70s and 80s. The increase in LS was gradual in middle-aged individuals; however, it was rapid in older adults. Trajectories of decrease in mobility might differ by sex, especially among the middle-aged population.

Low physical performance, as detected via usual walking speed, sit-to-stand ability, and one-leg standing time, has been reported as a risk factor for LS in the dwelling cohort [10,22]. Only one study has reported risk factors for LS in patients with cancer [5]. Hirahata et al. reported that old age (≥65 years) and being underweight (<18.5 kg/m^2^) were independent risk factors for LS stage 3 in patients with cancers of various origins and stages. In this study, we focused on preoperative patients with localized cancer and identified age (≥60 years), grip strength, and %VC as risk factors associated with LS. The association between age and LS has been reported in a study conducted on community-dwelling residents [15]. Increasing age is a main cause of decreased mobility, aside from other predictors, including socioeconomic status, physical activities, or chronic conditions [23,24]. Age-dependent decrease in mobility has been reported among community dwellers in previous studies [14,23-25]. In this study, we discovered that age has a remarkable effect on the incidence rate of LS. The rate of LS gradually increased in middle-aged individuals but rapidly increased in older adults.

In patients with advanced cancer, grip strength is associated with malnutrition, altered cognitive function, and poor prognosis after surgery [26,27]. Kobayashi reported that grip strength was significantly associated with the GLFS-25 total score and the diagnosis of LS in healthy Japanese older adults who attended the annual public health checkup. In this study, the rates of LS were 99% and 86% in patients with or within criteria of grip strength, respectively. Based on these previous reports, grip strength affects LS and can be a candidate marker of potential LS [28,29]. The rate of LS was significantly higher in gastric cancer than in several other cancers when analyzing LS stages 2 and 3. The reason for this may be that patients with gastric cancer are older than patients with other cancer types and, therefore, have a higher proportion of LS.

This study has several limitations. First, it focused on perioperative patients with cancer, but the cancer types were limited due to the institutional referral pattern. Patients with breast and bladder cancers were excluded because these cancers are frequently treated with minimally invasive procedures, which fall outside the scope of our perioperative rehabilitation program designed for major surgeries. Another important limitation is the absence of postoperative outcome data, which precludes an evaluation of how preoperative LS impacts recovery after surgery. Future longitudinal studies are necessary to assess the clinical implications of LS in cancer care. In addition, subgroup analyses were limited by small sample sizes for certain cancer types, which may affect generalizability. We also acknowledge the potential influence of unmeasured confounders. Prospective and interventional studies are warranted to further investigate the long-term impact of LS and to develop strategies for early intervention. Additionally, we investigated disability only through LS tests, as we did not use other methods, such as sarcopenia, in all patients. Acquiring an exercise habit, ensuring appropriate nutrition, being active, and evaluating and treating locomotion-related diseases are important for delaying or avoiding LS.

## Conclusions

The overall rate of LS was as high as 88.1%, with a gradual increase in prevalence from middle-aged to older individuals. Age, reduced grip strength, and decreased vital capacity were identified as associated factors. Clinicians should be aware of this high prevalence among preoperative patients with localized cancer. While our findings suggest a possible association between preoperative LS and postoperative recovery, this interpretation is hypothesis-generating and warrants future longitudinal research.

## References

[1] Zugazagoitia J, Guedes C, Ponce S, Ferrer I, Molina-Pinelo S, Paz-Ares L (2016). Current challenges in cancer treatment. Clin Ther.

[2] Fitch MI, Nicoll I, Newton L, Strohschein FJ (2022). Challenges of survivorship for older adults diagnosed with cancer. Curr Oncol Rep.

[3] Morishita S, Tsubaki A, Fu JB, Mitobe Y, Onishi H, Tsuji T (2018). Cancer survivors exhibit a different relationship between muscle strength and health-related quality of life/fatigue compared to healthy subjects. Eur J Cancer Care (Engl).

[4] Tsuji T (2022). Rehabilitation for elderly patients with cancer. Jpn J Clin Oncol.

[5] Hirahata M, Imanishi J, Fujinuma W (2023). Cancer may accelerate locomotive syndrome and deteriorate quality of life: a single-centre cross-sectional study of locomotive syndrome in cancer patients. Int J Clin Oncol.

[6] Kawano H, Hirahata M, Imanishi J (2022). Locomotive syndrome in cancer patients: a new role of orthopaedic surgeons as a part of comprehensive cancer care. Int J Clin Oncol.

[7] Sato M, Furuya T, Shiga Y (2022). Assessment of locomotive syndrome in patients with visceral cancer, the comparison with non-cancer patients using propensity score matching. J Orthop Sci.

[8] Seichi A, Hoshino Y, Doi T, Akai M, Tobimatsu Y, Iwaya T (2012). Development of a screening tool for risk of locomotive syndrome in the elderly: the 25-question Geriatric Locomotive Function Scale. J Orthop Sci.

[9] Ogata T, Muranaga S, Ishibashi H (2015). Development of a screening program to assess motor function in the adult population: a cross-sectional observational study. J Orthop Sci.

[10] Yoshimura N, Muraki S, Nakamura K, Tanaka S (2017). Epidemiology of the locomotive syndrome: the research on osteoarthritis/osteoporosis against disability study 2005-2015. Mod Rheumatol.

[11] Yoshimura N, Nakamura K (2016). Epidemiology of locomotive organ disorders and symptoms: an estimation using the population-based cohorts in Japan. Clin Rev Bone Miner Metab.

[12] Yoshimura N, Muraki S, Oka H (2015). Association between new indices in the locomotive syndrome risk test and decline in mobility: third survey of the ROAD study. J Orthop Sci.

[13] Yoshimura N, Oka H, Muraki S (2011). Reference values for hand grip strength, muscle mass, walking time, and one-leg standing time as indices for locomotive syndrome and associated disability: the second survey of the ROAD study. J Orthop Sci.

[14] Otani K, Takegami M, Fukumori N (2012). Locomotor dysfunction and risk of cardiovascular disease, quality of life, and medical costs: design of the Locomotive Syndrome and Health Outcome in Aizu Cohort Study (LOHAS) and baseline characteristics of the study population. J Orthop Sci.

[15] Yamada K, Ito YM, Akagi M (2020). Reference values for the locomotive syndrome risk test quantifying mobility of 8681 adults aged 20-89 years: a cross-sectional nationwide study in Japan. J Orthop Sci.

[16] Yasuhara T, Hishikawa T, Agari T (2016). Perioperative Management Center (PERIO) for neurosurgical patients. Neurol Med Chir (Tokyo).

[17] Chen LK, Woo J, Assantachai P (2020). Asian Working Group for sarcopenia: 2019 consensus update on sarcopenia diagnosis and treatment. J Am Med Dir Assoc.

[18] Kobayashi K, Imagama S, Ando K (2019). Locomotive syndrome stage 1 predicts significant worsening of future motor performance: the prospective Yakumo Study. Biomed Res Int.

[19] Yoshimura Y, Ishijima M, Ishibashi M (2019). A nationwide observational study of locomotive syndrome in Japan using the ResearchKit: the Locomonitor study. J Orthop Sci.

[20] Kimura A, Seichi A, Konno S, Yabuki S, Hayashi K (2014). Prevalence of locomotive syndrome in Japan: a nationwide, cross-sectional Internet survey. J Orthop Sci.

[21] Seichi A, Kimura A, Konno S, Yabuki S (2016). Epidemiologic survey of locomotive syndrome in Japan. J Orthop Sci.

[22] Nakamura M, Hashizume H, Oka H (2015). Physical performance measures associated with locomotive syndrome in middle-aged and older Japanese women. J Geriatr Phys Ther.

[23] Rantakokko M, Mänty M, Rantanen T (2013). Mobility decline in old age. Exerc Sport Sci Rev.

[24] House JS, Lepkowski JM, Kinney AM, Mero RP, Kessler RC, Herzog AR (1994). The social stratification of aging and health. J Health Soc Behav.

[25] Yamada K, Muranaga S, Shinozaki T, Nakamura K, Tanaka S, Ogata T (2018). Age independency of mobility decrease assessed using the Locomotive Syndrome Risk Test in elderly with disability: a cross-sectional study. BMC Geriatr.

[26] Pereira AA, Zaia RD, Souza GH, Luizeti BO, Andreola R, Junior AO, Ferrari A (2021). The correlation between hand grip strength and nutritional variables in ambulatory cancer patients. Nutr Cancer.

[27] Matsui R, Inaki N, Tsuji T (2021). The impact of the preoperative hand grip strength on the long-term outcomes after gastrectomy for advanced gastric cancer. Surg Today.

[28] Kobayashi T, Morimoto T, Ono R, Otani K, Mawatari M (2023). Is grip strength useful in screening to predict the severity of locomotive syndrome?. J Orthop Sci.

[29] Kobayashi K, Imagama S, Ando K (2020). Weakness of grip strength reflects future locomotive syndrome and progression of locomotive risk stage: a 10-year longitudinal cohort study. Mod Rheumatol.

